# *N*-[4-(2-Propyn-1-yl­oxy)phen­yl]acetamide

**DOI:** 10.1107/S1600536812041207

**Published:** 2012-10-06

**Authors:** Yonas H. Belay, Henok H. Kinfe, Alfred Muller

**Affiliations:** a Research Centre for Synthesis and Catalysis, Department of Chemistry, University of Johannesburg (APK Campus), PO Box 524, Auckland Park, Johannesburg, 2006, South Africa

## Abstract

The title compound, C_11_H_11_NO_2_, was synthesized by chemoselective *N*-acetyl­ation of 4-amino­phenol followed by reaction with propargyl bromide in the presence of K_2_CO_3_. the acetamide and propyn-1-yloxy substituents form dihedral angles of 18.31 (6) and 7.01 (10)°, respectively, with the benzene ring. In the crystal, mol­ecules are linked by N—H⋯O hydrogen bonds into chains along [010]. C—H⋯O and C—H⋯π inter­actions also occur.

## Related literature

For background to the development of hybrid drug candidates against tuberculosis, malaria and cancer, see: Morphy *et al.* (2004 ▶). For details of the synthesis of the title compound, see: Hoogendoorn *et al.* (2011 ▶); Reppe (1955 ▶).
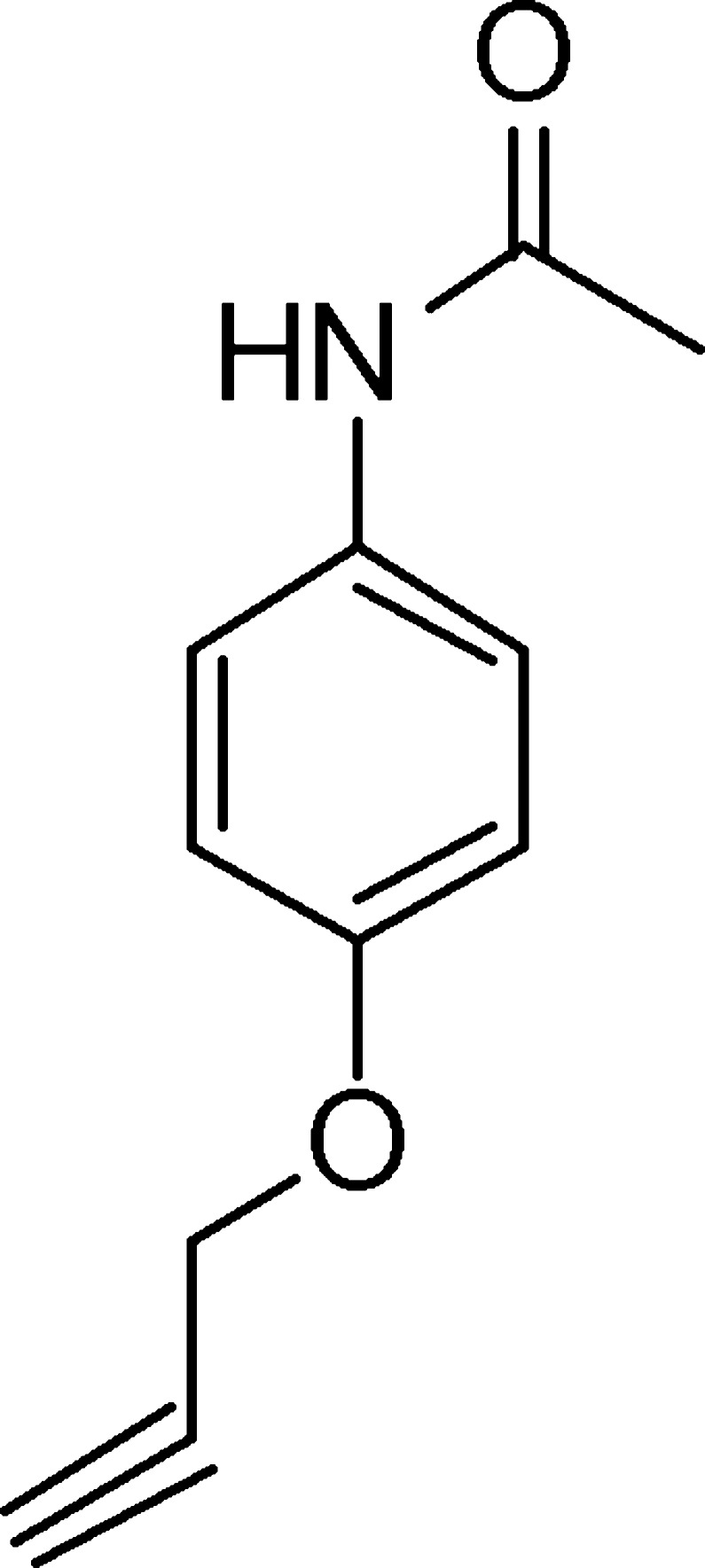


## Experimental

### 

#### Crystal data

C_11_H_11_NO_2_
*M* *_r_* = 189.21
Monoclinic, 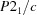

*a* = 13.973 (2) Å
*b* = 9.1794 (13) Å
*c* = 7.5105 (11) Å
β = 99.441 (4)°
*V* = 950.3 (2) Å^3^
*Z* = 4
Mo *K*α radiation
μ = 0.09 mm^−1^
*T* = 100 K
0.3 × 0.26 × 0.23 mm

#### Data collection

Bruker APEX DUO 4K CCD diffractometer
Absorption correction: multi-scan (*SADABS*; Bruker, 2008 ▶) *T*_min_ = 0.969, *T*_max_ = 0.981
6753 measured reflections
2388 independent reflections
2103 reflections with *I* > 2σ(*I*)
*R*_int_ = 0.018

#### Refinement

*R*[*F* ^2^ > 2σ(*F* ^2^)] = 0.037
*wR*(*F* ^2^) = 0.100
*S* = 1.04
2388 reflections
131 parameters
H atoms treated by a mixture of independent and constrained refinement
Δρ_max_ = 0.28 e Å^−3^
Δρ_min_ = −0.24 e Å^−3^

### 

Data collection: *APEX2* (Bruker, 2011 ▶); cell refinement: *SAINT* (Bruker, 2008 ▶); data reduction: *SAINT* and *XPREP* (Bruker, 2008 ▶); program(s) used to solve structure: *SIR97* (Altomare *et al.*, 1999 ▶); program(s) used to refine structure: *SHELXL97* (Sheldrick, 2008 ▶); molecular graphics: *DIAMOND* (Brandenburg & Putz, 2005 ▶); software used to prepare material for publication: *WinGX* (Farrugia, 1999 ▶).

## Supplementary Material

Crystal structure: contains datablock(s) global, I. DOI: 10.1107/S1600536812041207/nc2291sup1.cif

Structure factors: contains datablock(s) I. DOI: 10.1107/S1600536812041207/nc2291Isup2.hkl

Additional supplementary materials:  crystallographic information; 3D view; checkCIF report

## Figures and Tables

**Table 1:** Hydrogen-bond geometry (Å, °) *Cg*1 is the centroid of the C4–C11 ring.

*D*—H⋯*A*	*D*—H	H⋯*A*	*D*⋯*A*	*D*—H⋯*A*
N1—H1⋯O2^i^	0.879 (14)	1.993 (14)	2.8695 (11)	175.2 (13)
C9—H9*B*⋯O2^i^	0.98	2.53	3.4043 (14)	148
C5—H5⋯*Cg*1^ii^	0.95	2.69	3.5171 (12)	146
C9—H9*A*⋯*Cg*1^iii^	0.98	2.94	3.7373 (12)	139

Symmetry codes: (i) 
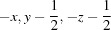
; (ii) 
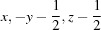
; (iii) 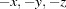.

**Fig. 1.**
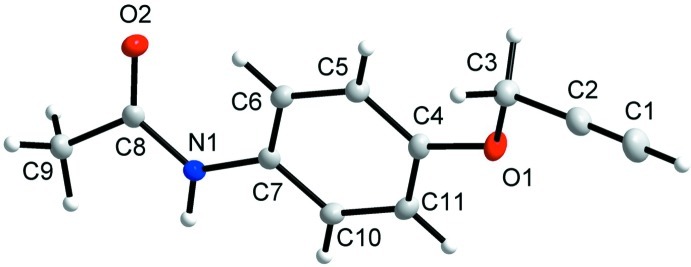
Crystal structure of the title compound with labeling and displacement ellipsoids drawn at the 50% probability level..

**Fig. 2.**
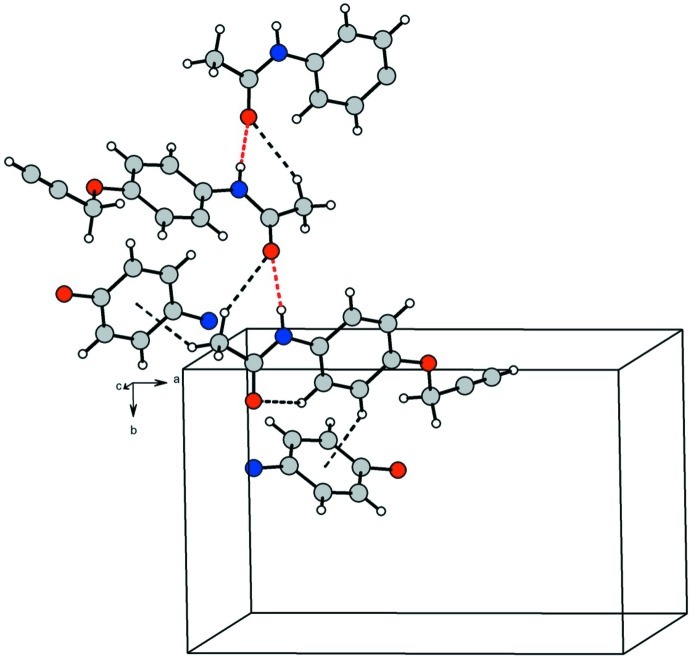
Packing diagram showing the molecules linked by infinite one-dimensional N—H···O chains in the [010] direction. Hydrogen bonds and C—H···O/π interactions are shown as red and black dashed lines respectively.

## supplementary crystallographic information

### Comment

In our pursuit in the development of hybrid drug candidates against tuberculosis, malaria and cancer (Morphy *et al.*, 2004), the title compound was identified as a building starting material. The compound was synthesized by chemoselective *N*-acetylation of 4-aminophenol followed by reaction with propargyl bromide in the presence of K_2_CO_3_ (Hoogendoorn *et al.*, 2011). To confirm the chemoselectivity, herein we report the single-crystal structure of the title compound.

In the crystal structure of the title compound the acetamide and propyn-1-yloxy substituents form dihedral angles to the six-membered ring of 18.31 (6)° and 7.01 (10)° respectively (Figure 1). Molecules are linked by infinite one-dimensional N—H···O hydrogen bonding into chains that elongate in the [010] direction (see Figure 2 for a visual summary).

### Experimental

A solution of *N*-(hydoxy-phenyl)acetamide (450 mg, 2.980 mmol), synthesized by chemoselective acetylation of 4-aminophenol using 1 equivalence of acetic anhydride, in dry acetone was treated with potassium carbonate (576 mg, 4.17 mmol). The reaction mixture was stirred under reflux for about 30 minutes followed by addition of propargyl bromide (0.8 ml, 6.56 mmol). The combined solution was stirred for additional 3 h and concentrated under *vacuo*. The residue was diluted with water and extracted three times with ethyl acetate. The combined organic layer was washed with brine and water and dried over anhydrous sodium sulfate, filtered and solvent evaporated. The solid crude product was recrystallized from dichloromethane and hexane to afford 71% of the target compound as pale yellow crystals. The melting point of the crystalline material was found to similar as reported in literature (Reppe, 1955).

### Refinement

All hydrogen atoms were positioned in geometrically idealized positions with C—H = 0.99 Å (methylene), 0.98 Å (methyl) and 0.95 Å (aromatic and acetylenic). The amide hydrogen atom were obtained from a Fourier difference map and refined with varying coordinates. All these hydrogen atoms were allowed to ride on their parent atoms with *U*_iso_(H) = 1.2*U*_eq_, except for methyl and amide hydrogen atoms where *U*_iso_(H) = 1.5*U*_eq_ was utilized. The initial positions of methyl hydrogen atoms were located from a Fourier difference map and refined as a fixed rotor.

### Crystal data

**Table:**

C_11_H_11_NO_2_	*F*(000) = 400
*M**_r_* = 189.21	*D*_x_ = 1.323 Mg m^−^^3^
Monoclinic, *P*2_1_/*c*	Mo *K*α radiation, λ = 0.71073 Å
Hall symbol: -P 2ybc	Cell parameters from 3099 reflections
*a* = 13.973 (2) Å	θ = 2.7–28.4°
*b* = 9.1794 (13) Å	µ = 0.09 mm^−^^1^
*c* = 7.5105 (11) Å	*T* = 100 K
β = 99.441 (4)°	Block, pale yellow
*V* = 950.3 (2) Å^3^	0.3 × 0.26 × 0.23 mm
*Z* = 4	

### Data collection

**Table:**

Bruker APEX DUO 4K CCD diffractometer	2388 independent reflections
Graphite monochromator	2103 reflections with *I* > 2σ(*I*)
Detector resolution: 8.4 pixels mm^-1^	*R*_int_ = 0.018
φ and ω scans	θ_max_ = 28.5°, θ_min_ = 2.7°
Absorption correction: multi-scan (*SADABS*; Bruker, 2008)	*h* = −18→18
*T*_min_ = 0.969, *T*_max_ = 0.981	*k* = −12→11
6753 measured reflections	*l* = −10→10

### Refinement

**Table:**

Refinement on *F*^2^	Primary atom site location: structure-invariant direct methods
Least-squares matrix: full	Secondary atom site location: difference Fourier map
*R*[*F*^2^ > 2σ(*F*^2^)] = 0.037	Hydrogen site location: inferred from neighbouring sites
*wR*(*F*^2^) = 0.100	H atoms treated by a mixture of independent and constrained refinement
*S* = 1.04	*w* = 1/[σ^2^(*F*_o_^2^) + (0.0509*P*)^2^ + 0.3049*P*] where *P* = (*F*_o_^2^ + 2*F*_c_^2^)/3
2388 reflections	(Δ/σ)_max_ < 0.001
131 parameters	Δρ_max_ = 0.28 e Å^−^^3^
0 restraints	Δρ_min_ = −0.24 e Å^−^^3^

### Special details

**Table:**

Experimental. The intensity data was collected on a Bruker Apex DUO 4 K CCD diffractometer using an exposure time of 20 s/frame. A total of 735 frames were collected with a frame width of 0.5° covering up to θ = 28.47° with 99.3% completeness accomplished. Analytical data: mp: 110 - 111 °C (Lit. 109 - 111 °C; Reppe, 1955); ^1^H NMR (CDCl_3_, 400 MHz): d 7.57 (s, 1H), 7.38 (d, *J* = 8.4 Hz, 2H), 6.89 (d, *J* = 8.8 Hz, 2H), 4.63 (s, 2H), 2.49 (s, 1H), 2.12 (s, 3H); ^13^C NMR (CDCl_3_,400 MHz): d 168.4, 154.3, 131.9, 121.8, 115.3, 78.5, 75.5, 56.1, 24.4.
Geometry. All e.s.d.'s (except the e.s.d. in the dihedral angle between two l.s. planes) are estimated using the full covariance matrix. The cell e.s.d.'s are taken into account individually in the estimation of e.s.d.'s in distances, angles and torsion angles; correlations between e.s.d.'s in cell parameters are only used when they are defined by crystal symmetry. An approximate (isotropic) treatment of cell e.s.d.'s is used for estimating e.s.d.'s involving l.s. planes.
Refinement. Refinement of *F*^2^ against ALL reflections. The weighted *R*-factor *wR* and goodness of fit *S* are based on *F*^2^, conventional *R*-factors *R* are based on *F*, with *F* set to zero for negative *F*^2^. The threshold expression of *F*^2^ > σ(*F*^2^) is used only for calculating *R*-factors(gt) *etc*. and is not relevant to the choice of reflections for refinement. *R*-factors based on *F*^2^ are statistically about twice as large as those based on *F*, and *R*- factors based on ALL data will be even larger.

### Fractional atomic coordinates and isotropic or equivalent isotropic displacement parameters (Å^2^)

**Table:**

	*x*	*y*	*z*	*U*_iso_*/*U*_eq_	
O1	0.44065 (5)	0.09706 (8)	0.16260 (10)	0.01982 (18)	
O2	−0.00853 (5)	0.27539 (8)	−0.14889 (10)	0.02019 (18)	
N1	0.05791 (6)	0.05346 (9)	−0.18684 (12)	0.01555 (19)	
C1	0.63468 (9)	0.09825 (14)	0.49415 (17)	0.0288 (3)	
H1A	0.6943	0.0641	0.5601	0.035*	
C2	0.56036 (8)	0.14084 (12)	0.41199 (15)	0.0215 (2)	
C3	0.46720 (8)	0.19131 (12)	0.31532 (14)	0.0202 (2)	
H3A	0.4174	0.1874	0.395	0.024*	
H3B	0.4726	0.2931	0.2748	0.024*	
C4	0.34482 (7)	0.09626 (11)	0.08432 (13)	0.0160 (2)	
C5	0.27561 (7)	0.19574 (11)	0.12113 (13)	0.0165 (2)	
H5	0.2934	0.2712	0.2068	0.02*	
C6	0.17996 (7)	0.18471 (11)	0.03218 (13)	0.0160 (2)	
H6	0.1327	0.2524	0.0582	0.019*	
C7	0.15352 (7)	0.07514 (10)	−0.09429 (13)	0.0143 (2)	
C8	−0.01572 (7)	0.15035 (11)	−0.20997 (13)	0.0156 (2)	
C9	−0.10956 (7)	0.09587 (11)	−0.31599 (14)	0.0192 (2)	
H9A	−0.1605	0.1005	−0.2407	0.029*	
H9B	−0.1014	−0.0051	−0.353	0.029*	
H9C	−0.1279	0.1568	−0.4233	0.029*	
C10	0.22426 (7)	−0.02335 (11)	−0.13094 (14)	0.0169 (2)	
H10	0.207	−0.0982	−0.2177	0.02*	
C11	0.31883 (7)	−0.01321 (11)	−0.04271 (14)	0.0175 (2)	
H11	0.3661	−0.0809	−0.0687	0.021*	
H1	0.0464 (10)	−0.0325 (16)	−0.2373 (18)	0.021*	

### Atomic displacement parameters (Å^2^)

**Table:**

	*U*^11^	*U*^22^	*U*^33^	*U*^12^	*U*^13^	*U*^23^
O1	0.0138 (4)	0.0255 (4)	0.0184 (4)	0.0032 (3)	−0.0024 (3)	−0.0051 (3)
O2	0.0192 (4)	0.0149 (3)	0.0248 (4)	0.0026 (3)	−0.0015 (3)	−0.0008 (3)
N1	0.0139 (4)	0.0124 (4)	0.0192 (4)	−0.0008 (3)	−0.0006 (3)	−0.0010 (3)
C1	0.0196 (6)	0.0338 (6)	0.0302 (6)	−0.0025 (5)	−0.0039 (5)	0.0011 (5)
C2	0.0192 (5)	0.0236 (5)	0.0211 (5)	−0.0036 (4)	0.0014 (4)	−0.0027 (4)
C3	0.0190 (5)	0.0213 (5)	0.0189 (5)	0.0002 (4)	−0.0010 (4)	−0.0032 (4)
C4	0.0136 (5)	0.0190 (5)	0.0148 (4)	0.0012 (4)	0.0000 (4)	0.0021 (3)
C5	0.0168 (5)	0.0165 (4)	0.0154 (4)	0.0005 (4)	0.0003 (4)	−0.0022 (3)
C6	0.0162 (5)	0.0150 (4)	0.0168 (5)	0.0020 (4)	0.0022 (4)	0.0000 (3)
C7	0.0140 (5)	0.0138 (4)	0.0146 (4)	−0.0001 (3)	0.0007 (3)	0.0027 (3)
C8	0.0146 (5)	0.0156 (4)	0.0162 (4)	−0.0001 (3)	0.0014 (4)	0.0032 (3)
C9	0.0147 (5)	0.0184 (5)	0.0234 (5)	−0.0007 (4)	−0.0006 (4)	0.0018 (4)
C10	0.0181 (5)	0.0139 (4)	0.0179 (5)	0.0004 (4)	0.0006 (4)	−0.0017 (3)
C11	0.0171 (5)	0.0159 (4)	0.0191 (5)	0.0040 (4)	0.0016 (4)	−0.0002 (4)

### Geometric parameters (Å, º)

**Table:**

O1—C4	1.3713 (12)	C5—C6	1.3965 (14)
O1—C3	1.4360 (12)	C5—H5	0.95
O2—C8	1.2340 (12)	C6—C7	1.3911 (13)
N1—C8	1.3495 (13)	C6—H6	0.95
N1—C7	1.4152 (13)	C7—C10	1.3999 (14)
N1—H1	0.879 (14)	C8—C9	1.5036 (14)
C1—C2	1.1840 (17)	C9—H9A	0.98
C1—H1A	0.95	C9—H9B	0.98
C2—C3	1.4582 (15)	C9—H9C	0.98
C3—H3A	0.99	C10—C11	1.3807 (14)
C3—H3B	0.99	C10—H10	0.95
C4—C5	1.3903 (14)	C11—H11	0.95
C4—C11	1.3922 (14)		
			
C4—O1—C3	116.86 (8)	C5—C6—H6	119.8
C8—N1—C7	127.50 (9)	C6—C7—C10	118.96 (9)
C8—N1—H1	117.0 (9)	C6—C7—N1	124.10 (9)
C7—N1—H1	115.5 (9)	C10—C7—N1	116.92 (9)
C2—C1—H1A	180	O2—C8—N1	123.46 (9)
C1—C2—C3	178.14 (12)	O2—C8—C9	121.13 (9)
O1—C3—C2	107.39 (8)	N1—C8—C9	115.41 (9)
O1—C3—H3A	110.2	C8—C9—H9A	109.5
C2—C3—H3A	110.2	C8—C9—H9B	109.5
O1—C3—H3B	110.2	H9A—C9—H9B	109.5
C2—C3—H3B	110.2	C8—C9—H9C	109.5
H3A—C3—H3B	108.5	H9A—C9—H9C	109.5
O1—C4—C5	124.98 (9)	H9B—C9—H9C	109.5
O1—C4—C11	115.11 (9)	C11—C10—C7	120.87 (9)
C5—C4—C11	119.90 (9)	C11—C10—H10	119.6
C4—C5—C6	119.98 (9)	C7—C10—H10	119.6
C4—C5—H5	120	C10—C11—C4	119.94 (9)
C6—C5—H5	120	C10—C11—H11	120
C7—C6—C5	120.34 (9)	C4—C11—H11	120
C7—C6—H6	119.8		
			
C4—O1—C3—C2	161.20 (9)	C8—N1—C7—C10	−162.01 (10)
C3—O1—C4—C5	11.37 (14)	C7—N1—C8—O2	−0.68 (16)
C3—O1—C4—C11	−169.65 (9)	C7—N1—C8—C9	179.68 (9)
O1—C4—C5—C6	179.65 (9)	C6—C7—C10—C11	0.40 (15)
C11—C4—C5—C6	0.72 (15)	N1—C7—C10—C11	−177.99 (9)
C4—C5—C6—C7	−0.44 (15)	C7—C10—C11—C4	−0.13 (15)
C5—C6—C7—C10	−0.12 (14)	O1—C4—C11—C10	−179.47 (9)
C5—C6—C7—N1	178.15 (9)	C5—C4—C11—C10	−0.44 (15)
C8—N1—C7—C6	19.69 (15)		

### Hydrogen-bond geometry (Å, º)

Cg1 is the centroid of the C4–C11 ring.

**Table:**

*D*—H···*A*	*D*—H	H···*A*	*D*···*A*	*D*—H···*A*
N1—H1···O2^i^	0.879 (14)	1.993 (14)	2.8695 (11)	175.2 (13)
C6—H6···O2	0.95	2.31	2.8816 (13)	118
C9—H9*B*···O2^i^	0.98	2.53	3.4043 (14)	148
C5—H5···*Cg*1^ii^	0.95	2.69	3.5171 (12)	146
C9—H9*A*···*Cg*1^iii^	0.98	2.94	3.7373 (12)	139

Symmetry codes: (i) −*x*, *y*−1/2, −*z*−1/2; (ii) *x*, −*y*−1/2, *z*−1/2; (iii) −*x*, −*y*, −*z*.
